# Immunogenicity of Immunotoxins Containing *Pseudomonas* Exotoxin A: Causes, Consequences, and Mitigation

**DOI:** 10.3389/fimmu.2020.01261

**Published:** 2020-06-26

**Authors:** Ronit Mazor, Ira Pastan

**Affiliations:** ^1^Division of Cellular and Gene Therapies, Center for Biologics Evaluation and Research, Food and Drug Administration, Silver Spring, MD, United States; ^2^Laboratory of Molecular Biology, Center for Cancer Research, National Cancer Institute, National Institutes of Health, Bethesda, MD, United States

**Keywords:** recombinant immunotoxins, neutralizing antibodies, anti-drug antibodies (ADA), B cell epitopes, T cell epitopes, moxetumomab pasudotox, LMB-100

## Abstract

Immunotoxins are cytolytic fusion proteins developed for cancer therapy, composed of an antibody fragment that binds to a cancer cell and a protein toxin fragment that kills the cell. *Pseudomonas* exotoxin A (PE) is a potent toxin that is used for the killing moiety in many immunotoxins. Moxetumomab Pasudotox (Lumoxiti) contains an anti-CD22 Fv and a 38 kDa portion of PE. Lumoxiti was discovered in the Laboratory of Molecular Biology at the U.S. National Cancer Institute and co-developed with Medimmune/AstraZeneca to treat hairy cell leukemia. In 2018 Lumoxiti was approved by the US Food and Drug Administration for the treatment of drug-resistant Hairy Cell Leukemia. Due to the bacterial origin of the killing moiety, immunotoxins containing PE are highly immunogenic in patients with normal immune systems, but less immunogenic in patients with hematologic malignancies, whose immune systems are often compromised. LMB-100 is a de-immunized variant of the toxin with a humanized antibody that targets mesothelin and a PE toxin that was rationally designed for diminished reactivity with antibodies and B cell receptors. It is now being evaluated in clinical trials for the treatment of mesothelioma and pancreatic cancer and is showing somewhat diminished immunogenicity compared to its un modified parental counterpart. Here we review the immunogenicity of the original and de-immunized PE immunotoxins in mice and patients, the development of anti-drug antibodies (ADAs), their impact on drug availability and their effect on clinical efficacy. Efforts to mitigate the immunogenicity of immunotoxins and its impact on immunogenicity will be described including rational design to identify, remove, or suppress B cell or T cell epitopes, and combination of immunotoxins with immune modulating drugs.

## Introduction

Protein and cell based therapeutic agents have great potential to treat many human diseases. However, because many of these contain non-self sequences, they often elicit an immune response that blocks their efficacy. Clinical trials with chimeric antigen receptor-T cells (CAR-T) (1), enzyme replacement therapy (2), monoclonal antibodies (3), antibody drug conjugates (ADCs), immunotoxins (4), and viral based gene therapy vectors (5) have often failed to produce desired effects due to the formation of antibodies that neutralize the activity of the therapeutic agent.

Recombinant immunotoxins (RIT) are chimeric proteins that consist of a targeting element linked to a toxin. The targeting element is commonly an Fv portion of an antibody which targets a specific antigen on tumor or infected cells (6). RITs have been developed to treat a variety of indications, such as blood cancers (7, 8) solid tumors (9–11) graft-vs.-host disease (12), viral infections (13, 14), and autoimmune diseases (15). *Pseudomonas* exotoxin A (PE, also known as ETA) and diphtheria toxin are both favorable toxins for construction of RITs due to their high potency, expression and purification yields, ease of cloning, and relatively low non-specific toxicity compared to other toxins (16). Both toxins kill cells by catalyzing ADP ribosylation and inactivation of elongation factor 2, which results in arrest of protein translation, a fall in anti apoptotic proteins and apoptosis (11). Both toxins have been used as killing domains in antibody or cytokine targeted drugs and were approved for licensure by regulatory agencies. They represent “first in class” drugs for targeted toxins (17, 18).

Recently (September 2018), Moxetumomab pasudotox (Lumoxiti), whose pre-clinical and early clinical development took place in the Laboratory of Molecular Biology (LMB) at the U.S. National Cancer Institute and whose advanced clinical development took place at AstraZeneca, was approved by the U.S. Food and Drug Administration for the treatment of relapsed or refractory hairy cell leukemia. Lumoxiti is composed of an anti-CD22 Fv murine antibody fused to PE38, a 38 kDa truncated form of PE (Table 1) (26, 27). Encouraged by this success, major efforts are focused on developing PE based RITs against mesothelin and other proteins on solid tumors (20, 28–32).

**Table 1:** Immunotoxins tested in patients.

**RIT name**	**Target**	**Antibody clone**	**Antibody format**	**Mouse or human**	**Clinical trial**	**References**
D2C7-(scdv)-PE38KDEL	EGFR	D2C7	scdsFv	Mouse	NCT02449239	(19)
LMB-1	Lewis Y	B3	Mab	Mouse	NCT00001805	(20)
Oportuzumab Monatox	Anti-EpCAM	VB4-845	scFv	Humanized	NCT03258593	(21)
Moxetumomab Pasudotox (Lumoxiti)	CD22	Affinity matured RFB4	scFv	Mouse	NCT01829711	(22)
LMB-2	CD25	Anti Tac	scFv	Mouse	NCT00924170	(23)
MOC31PE	EpCAM	MOC31	scFv	Mouse	NCT02219893	(21)
SS1P	Mesothelin	SS1	scdsFv	Mouse	NCT00006981	(24)
LMB-100	Mesothelin	SS1	Fab	Humanized	NCT02798536	(25)

## *Pseudomonas* Exotoxin a (PE)

PE is the most toxic virulence factor of the opportunistic pathogen *Pseudomonas aeruginosa* (33), a Gram-negative bacterium (34). *Pseudomonas aeruginosa* is ubiquitous in soil and water and generally infects only immunocompromised and elderly populations (34). This indicates that immune competent patients can efficiently mount an immune response and maintain an immune memory against *Pseudomonas aeruginosa* toxins. Indeed, the immunogenicity of the PE based moiety is a major hurdle in immunotoxin clinical development. PE is composed of three structural domains. A binding domain (I), a processing domain (II) and the catalytic domain (III). For RIT construction, the binding domain was replaced with antibody fragments.

## Methods to Assess Antibody Responses Against Recombinant Immunotoxins

Clinical development of RITs has been ongoing for about three decades. Immune monitoring of ADA by enzyme-linked immunosorbent assay (ELISA) or neutralizing antibodies (Nab) by neutralization assays (Nab assay) have changed in the past three decades as methods improved and as clinical development progressed. In early trials, ADAs were monitored using direct ELISA assays (28). A functional Nab assay was first reported in 1996 (20). The Nab assay entailed adding serum samples to two concentrations of immunotoxin and adding the mixture to sensitive cells. A sample was considered Nab positive if protein synthesis inhibition (20) or cytotoxic activity (7) was inhibited by 50 or 75%, respectively. Comparison of ADA positive patients and Nab positive assays revealed that all Nab positive samples are ADA positive but not all ADA positive samples are Nab positive (4). This indicates a higher sensitivity for the ADA assays and implies that some of the binding antibodies do not possess neutralization activity.

In the past decade, advancements in ADA monitoring methods and development of ultrasensitive assays have led to more specific and accurate monitoring approaches. Liang and colleague. improved the ELISA assay by minimizing the impact of PE38 immunodominance on the ability to detect ADA against the murine antibody fragment. They tested each patient's sample in a bridging ELISA with biotin-Lumoxiti coated plates in three conditions: with the CD22 fragment, with the PE38 fragment or with both. A signal was obtained using the addition of a ruthenium labeled Lumoxiti (35) and the fragments in the three conditions competed for binding with the ADAs.

To determine the immunogenicity cut-point for Lumoxiti (the OD or neutralization activity at which a sample is considered positive) samples from normal donors are commonly used. Because many naïve donors have been exposed to PE and have pre-existing antibodies, sample manipulation was necessary to obtain a sensitive cut-point for immunogenicity monitoring. To overcome this problem, an irrelevant PE-immunotoxin was added to serum samples to occupy the pre-existing antibodies prior to evaluating samples for cut point establishment (36).

## Clinical Immunogenicity of Immunotoxins

### Chemical Conjugates

**Figure 1:**
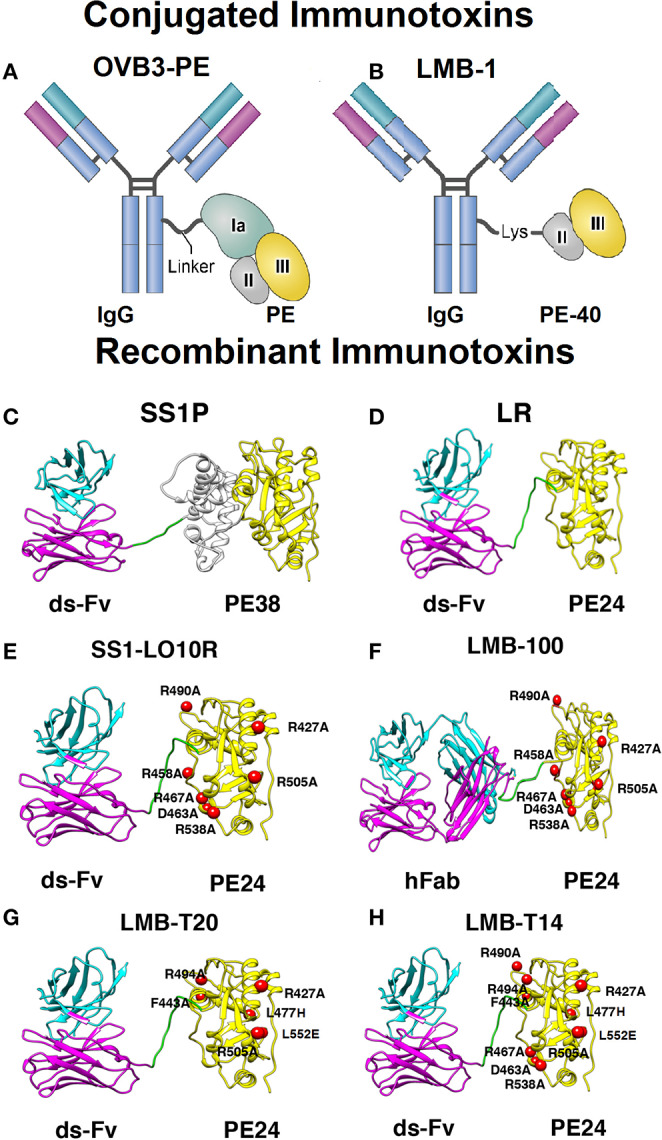
Models and structural models of conjugated and recombinant immunotoxins. **(A)** OVB3-PE is composed of a mouse IgG chemically conjugated to full length *Pseudomonas* exotoxin A (PE) using a linker. **(B)** LMB-1 consists of a mouse IgG chemically conjugated via a lysine residue to a 40 kDa fragment of PE that contains domain II (gray), domain III (yellow), and domain Ib (not shown). **(C)** SS1P consists of the disulfide-stabilized (ds) heavy chain Fv (V_H_; magenta) and light chain Fv (V_L_; cyan) of the antibody fragment SS1. The V_H_ is linked to PE38 that contains domains II and III but Ia is deleted. **(D)** Lysosome resistance (LR) immunotoxin. The ds-Fv of SS1P is linked to a 24-kDa fragment consisting of domain III of PE38 (termed PE24) **(E)** SS1-LO10R. A 24-kDa fragment of PE24 with 6-point mutations in domain III designed to suppress binding to B cell receptors. Point mutations are marked with red balls. **(F)** LMB-100 consists of a humanized Fab linked to LO10R PE24 toxin fragment and 6 point mutations as in E. **(G)** LMB-T20. PE24 with 6-point mutations in domain III designed to diminish T cell epitopes. **(H)** LMB-T14. PE24 with 10-point mutations in domain III designed to diminish B and T cell epitopes. All images are based on the structures of native PE and IgG. Images **(C–H)** were adapted from Mazor et al. (37).

The immunotoxin that was evaluated in a clinical trial (OVB3-PE) (Figure 1A) contained a full length murine antibody (clone OVB3) against an unknown antigen on ovarian cancer cells chemically conjugated to the PE protein (28). Formation of ADA against OVB3-PE was evaluated by ELISA, which showed that 16/16 of the patients developed anti PE ADA within 14 days. Human anti-mouse antibodies (HAMA) were also detected in 75% of the patients.

In a clinical trial evaluating LMB-1 (Figure 1B), in which domain I of PE was removed and the remaining 40 kDa protein attached to an antibody to Lewis-Y, 33/39 of the patients developed ADA responses against LMB-1 3 weeks after the first cycle of treatment. The remaining 10% who did not make neutralizing antibodies after the first cycle, were further treated and developed neutralizing antibodies after subsequent treatment cycles. ELISA assays indicated that eventually, 100% of the 38 patients made antibodies against the toxin moiety and 33/38 of the patients had HAMA against the antibody fragment (20).

### Recombinant Immunotoxins

LMB-2 (structure similar to SS1P shown in Figure 1C), which is composed of a murine anti CD-25 Fv linked to PE38 (Table 1), was used to treat leukemia and lymphoma patients. Clinical evaluation showed several complete and partial responses; however, 10/35 of the patients developed Nabs, which prevented further treatment. Six of the patients developed Nabs after the first cycle of treatment. Three of the patients that developed Nabs also demonstrated immunogenicity related side effects including one anaphylactic reaction and other allergic grade 2–3 reactions (2/35) (7). These adverse events contra-indicated further treatments once Nabs are present.

The lowest immunogenicity rates were reported in early trials evaluating Lumoxiti for hematological malignancies; After the first treatment cycle, only 1/28 hairy cell leukemia (HCL) patients made Nabs and a total of 10/28 had Nabs throughout the entire phase 1 trial (38). Furthermore, of the 50 CLL patients that were treated with Lumoxiti, only two patients had a Nab response after four cycles of treatment (4, 39).

Phase II and III trials of Lumoxiti were monitored for presence of binding ADA rather than Nabs. In those trials, 65% of patients made ADA after two cycles (38). In a larger trial, 75% of patients had detectable ADA at the end of treatment (40). The difference in immunogenicity reports in the early trials is mostly explained by differences in monitoring methods; functional Nab assays are less sensitive than binding ADA assays.

The overall low rate of immunogenicity to Lumoxiti can be attributed to the immune status of the patients. Patients with HCL have usually been treated with Cladribine which kills immune cells in the bone marrow. Additionally, the leukemia cells infiltrate the marrow, causing immunosuppression. Furthermore, Lumoxiti targets the CD22 antigen which is highly expressed in the targeted cancer cells but also expressed in mature and immature B cells. It is likely that Lumoxiti kills some B cells that would mount an immune response against it.

A good example that exemplifies the importance of patients' immune status is that of LMB-2. Patients with hematological malignancies treated with LMB-2 had a relatively low rate of immunogenicity onset with 17% of the patients making neutralizing antibodies after the first cycle (7). In contrast, melanoma patients who received LMB-2 and had a normal immune system demonstrated a high level of immune response; 92% of patients made neutralizing antibodies after the first cycle (41).

## Impact of Immunogenicity on Pharmacokinetics and Clinical Outcome

Generally, ADAs to therapeutic proteins have a risk of immune-related adverse events, including infusion-related reactions, allergic or anaphylactic reactions, delayed hypersensitivity, and autoimmunity (42). RITs show few of these responses. The only severe anaphylactic reaction reported occurred immediately after the first infusion of the RIT (7). Some patients reported grade 1, 2, or 3 skin reactions that were easily managed by a course of steroids [reviewed in (4)]. Neutralization and drug clearance are the main problems with RIT therapy, not immuno-toxicity. The low incidence of adverse side effects could be related to the relatively low doses administered and the small size (63 kDa) of the protein.

LMB-100 is a PE-based RIT engineered for decreased immunogenicity (Figure 1F). To study the impact of ADAs on LMB-100 levels, we analyzed immunogenicity and pharmacokinetic date from a clinical trial treating Pancreatic Ductal Adenocarcinoma with LMB-100 and nab-paclitaxel (25). Anti LMB-100 ADA were monitored using ultrasensitive methods to triage ADA positive and negative responses (screening assay). Patients with pre-existing antibodies were not excluded.

**Figure 2:**
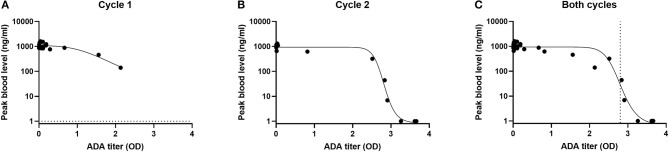
Impact of ADA on maximal concentration of LMB-100 (C_max_) in blood of patients. C_max_ and ADA measurements were performed as described in (25). C_max_ and ADA results were obtained from (25). C_max_ values were log transformed and fitted to an asymmetric sigmodial, 5-parameter curve fit. Dotted line represents EC_50_.

Using a cut point of O.D = 0.05, 9/20 had pre-existing antibodies. These low titers did not have much impact drug levels (Figure 2A). C_max_ in 19/20 patients was well above 100 ng/ml. In cycle 2, only 6/13 patients were ADA negative and their C_*max*_ well above 100 ng/ml. 7/13 of the patients were ADA positive still had effective blood levels (Figure 2B). Overall, more than half of patients receiving a second cycle of LMB-100 had detectable plasma drug concentrations. None of the patients received a third cycle of therapy due to toxicity of the nab-pactaxel. However, post treatment ADA monitoring showed that 9/10 of the patients evaluated were ADA positive; most of them with a very high OD signal (25).

Altogether, a correlation was observed between the ADA levels and drug blood levels (Figure 2C). Using a five-parameter asymmetric sigmoidal curve fit, EC50 has an OD = 2.8. Therefore, it can be estimated that samples with an OD lower than 2.8 will predict an effective C_max_ and samples with OD >than EC50 will not. Previously, patients with positive signals on ADA or Nab assays were excluded from clinical trials. However, this data indicates that a positive call on the ADA assay does not predict a low blood level unless the titers are very high (Figure 2).

Kreitman et al. reported that a minimum of three to five cycles of treatment was required to obtain major responses including durable complete remissions (43). In earlier trials, patients were not allowed to complete the therapy once they developed Nabs. This was to avoid immunological side effects unnecessary ineffective RIT drug administration. Kreitman and colleagues were able to observe a correlation between the timing of antibody formation and the outcome of the treatment (43). In the phase 1 study, 65% of patients made ADAs after two cycles based on ELISA results (38). Most patients (80%) who did not achieve CR had a positive antidrug antibody ELISA test (38). In a larger trial, patients with favorable responses (complete or partial responses) had lower antibody titers (<10,000), which probably improved their drug blood levels for more treatment cycles compared to patients with stable or progressive disease (40). Furthermore, when SS1P (Figure 1C) was combined with pentostatin and cyclophosphamide to lower T and B cells and suppress anti-drug antibodies, more treatment cycles could be given to most of the patients and major tumor responses were observed in several patients with advanced refractory mesothelioma (44). Altogether, these findings indicate that patients with low or delayed immune responses are likely to respond better and justifies the efforts described below to mitigate the ADA response.

## Strategies to Mitigate the Immunogenicity of RitS

### Combination With Immune Modulating Drugs

**Figure 3:**
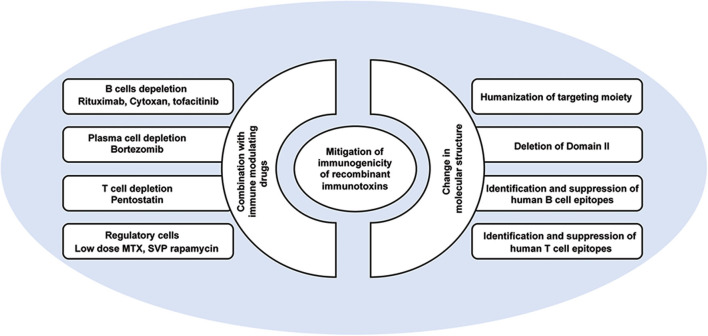
Approaches to mitigate immunogenicity of PE-based recombinant immunotoxins.

Combination approaches to mitigate immunogenicity of RIT include targeting of the B cells that form the adaptive immune response, targeting the plasma cells that produce high titers of IgG, or targeting the T cells that support a neutralizing immune response. In addition, recent approaches have targeted regulatory factors of the immune system that suppress the immune responses (Figure 3).

In 2004 five patients were pre-treated with rituximab to eliminate their B cells prior to LMB-1 administration. Binding ADA and Nab assay were used to monitor the development of human antibodies against LMB-1. Treatment with rituximab was effective in abrogating 99.9% of circulating CD20/CD19^+^ B cells in all patients (5/5). However, all these patients developed neutralizing anti-LMB-1 antibodies by day 21 of drug administration (45). This indicates that elimination of the peripheral B cells is not sufficient to eliminate the immune response.

To target both B and T cells, 10 refractory mesothelioma patients were treated with a combination of pentostatin and cyclophosphamide to kill B and T-cells. This combination delayed the formation of neutralizing antibodies to SS1P by several cycles. Out of the 10 patients treated, only two made Nabs after the first cycle, and 6 patients made Nabs after the second cycle. One patient did not make any Nabs throughout six treatment cycles (44). The toxicity observed in the trial described above was similar to the known side effects of pentostatin and cyclophosphamide.

In a T cell leukemia clinical trial evaluating the efficacy of LMB-2 after both cyclophosphamide and fludarabine, a great decrease in immunogenicity was observed. This delay translated to higher drug blood levels for multiple treatment cycles and very good anti-tumor responses (60% of the patients achieved complete remission) (23).

Elimination of pre-existing antibodies and plasma cells is a major goal for RITs due to a high prevalence of pre-existing antibodies from environmental exposure to PE, as well as the need to have more than one treatment cycles. Bortezomib is a reversible proteasome inhibitor that showed high efficacy in targeting long and short lived plasma cells that have high rates of Ig production (46). Manning et al. found that combination of SS1P with Bortezomib was able to reduce ADA formation by 50% compared to SS1P with no immune suppression in mice. Additional combination with pentostatin and cyclophosphamide reduced ADA formation by 88% (47).

Tofacitinib is a janus kinase 1 inhibitor that suppresses inflammatory responses. Treatment of mice with tofacitinib led to reduced numbers of CD127^+^ pro-B cells and reduction in B cell germinal center formation in mice spleens (48). Because normal Ig levels were still present during tofacitinib treatment, this agent specifically reduced ADAs.

Along with the immune depleting approaches, pre-clinical approaches to evaluate combinations with drug in low concentrations or encapsulated in nanoparticles have shown promising results. Low dose methotrexate (MTX) has been shown to reduce ADA formation against adalimumab [reviewed in (49, 50)] and against enzyme replacement therapy for infantile Pompe disease (51). Combination of low dose MTX with LMB-100 suppressed the formation of ADAs, maintained blood levels of LMB-100 and prevented its neutralization in immune competent mice. This did not compromise the immune response against a second antigen given after stopping MTX, suggesting contemporaneous immune tolerance (52).

To harness the immune modulatory properties of rapamycin, LMB collaborated with Selecta Bioscience that had encapsulated rapamycin in PLGA-PEG synthetic vaccine particles (SVP-R). Combination of SVP-R with LMB-100 produced a specific and transferable immune tolerance, which prevented ADA and Nab formation against the RIT in naïve mice and in mice that model pre-existing immunity (53). This approach was quickly translated to a clinical trial combining the two agents to treat mesothelioma patients. However, the combination resulted in an unforeseen lung toxicity in this patient population and the trial was discontinued (clinicaltrials.gov T03436732).

### Change in Molecular Structure

The two-unit structure of RITs which includes a targeting antibody unit and a toxin unit, and the variable immunogenicity properties of those two units (i.e., preexisting antibodies to the toxin or the presence of a murine fragment in the antibody) allows tailored mitigation to each unit based on its properties and what is known in the art as de-immunization (Figure 3).

## Mitigating the Immunogenicity of the Antibody Domain

Immunogenicity of therapeutic monoclonal antibodies can be mitigated by increasing the content of the human sequence. Such antibody engineering includes framework humanization, chimerization, and use of mice with humanized germlines. Such approaches can reduce the common immunogenicity rate from about 40% in chimeric antibodies to 9% in humanized antibodies (54, 55). However, in some cases, the immunogenicity against the variable complementarity determining region domains (CDRs) may still cause ADA formation (55).

Most RITs have a murine antibody fragment (Table 1). The lack of humanization of these agents can be explained by the fact that their development began before approaches to humanize antibodies were readily available. Furthermore, the immunogenicity of the bacterial PE is a much bigger barrier than HAMA (56). Recently, LMB-100 a “second generation RIT” containing a humanized Fab instead of a mouse Fv has entered clinical testing (Table 1) (25). The humanization was done by combining framework regions in the CDRs of the mouse anti-mesothelin antibody SS1 and human Fab. To improve the binding to mesothelin and to stabilize the CDRs tertiary structure, some back mutations within the mouse parent residues as well as the human sequences were introduced (as described in patent WO2015051199). The new humanized antibody had comparable binding affinity to mesothelin, and LMB-100 showed comparable thermal stability and technical developability to that of SS1P (57).

## Mitigating the Immunogenicity of the Toxin

### Identification of B Cell Epitopes

Antibodies and B-cell receptors bind to regions on the surface of a protein called B cell epitopes. These epitopes often cluster on the surface of the antigen and can control most immune responses (58). Roscoe et al. used synthetic peptides from PE38 to map the B cell epitopes in serum samples from monkeys and humans treated with immunotoxins (59, 60). This approach identified linear epitopes but not discontinuous conformational B cell epitopes on the toxin. Onda and Nagata immunized mice with RIT and used a capture assay to isolate monoclonal antibodies that reacted with native PE38 in solution (61). They discovered seven murine conformational epitopes in PE38 and identified single point alanine substitutions that abolished binding to those antibodies. They constructed and characterized a novel de-immunized mouse RIT named 8M. This RIT retained excellent cytotoxic and anti-tumor activity and importantly, had a low immunogenicity response after injection into mice. These experiments established the first proof that removing B cell epitopes could greatly diminish immunogenicity (61, 62).

Human B-cell epitopes in domain III were mapped using phage display. These studies focused on domain III of PE, because it was found that most of domain II was not needed to make active immunotoxins and could be removed (63). B cells were isolated from 7 patients receiving immunotoxin therapy and phage Fv libraries was prepared from B cells that contained Fvs reacting with domain III of PE. This selected library should represent the antibody repertoire that can bind and neutralize RITs with domain III. Then an immunotoxin library was constructed. This library contained 36 mutant PE immunotoxin constructs, each with a single point mutation replacing large amino acids like arginine, glutamine and glutamic acid with alanine. Then, the phage library was panned against each mutant RIT in the mutant library, identifying point mutations that abolish binding (64). Seven major B cell epitopes were identified and subsequently silenced by converting a key residue in the epitope to alanine. The modified toxin was named LO10 (Figure 1E) (representing the initials of the last name of the two scientists developing it). The LO10 toxin (Table 2) has been used to make immunotoxins targeting both CD22 and mesothelin. LMB-100 contains the LO10 mutations and is the first “de-immunized” PE based toxin that has advanced to clinical development.

**Table 2:** Recombinant Immunotoxins that were mutated to decrease immunogenicity.

**Drug name**	**Target**	**Toxin description**	**Activity**			**References**
			**IC50 (pM)**	**Relative activity (compared to PE38) (%)**	**Cell type**	
Moxetumomab	CD22	PE38	3.4	100	CA46	(65)
LO10		PE24 with 5 point mutations to reduce B cell binding	0.9	378		(64)
LMB-T18		PE24 with 6 point mutations to reduce T cell binding	2.2	155		(65)
LMB-T19		PE24 with 10 point mutations to reduce B and T cell binding	3.4	100		Unpublished
SS1P	Mesothelin	PE38	47.5	100	KLM1	(66)
LMB-100/RG7787		Humanized Fab and PE24 with 5 point mutations to reduce B cell binding	9.9	480		
LMB-T20		PE24 with 6 point mutations to reduce T cell binding	13.1	363		
LMB-T14		PE24 with 10 point mutations to reduce B and T cell binding	27.9	170		
LMB-2	CD25	PE38	0.07	100	HUT102	(67)
LMB-2 T20		PE38 with 6 point mutations to reduce T cell binding	0.23	30		
LMB-142		PE38 with 9 point mutations to reduce T cell binding	0.69	10		
Tac-M18-PE24(T)		PE24 with C-C stabilizing linker and 6 point mutations to reduce T cell binding	0.7	10		(68)
LMB-75	BCMA	PE24	1.1	100	H929	(69)
		PE24 with 4 point mutations to reduce B cell binding	3.1	35		Unpublished
LMB-92						
LMB-103 (T20)						
		PE24 with 6 point mutations to reduce T cell binding	6	18		Unpublished
LMB-273 (T20)		PE24 with 5 point mutations to reduce T cell binding (excluding R494A)	1.1	100		Unpublished
HN3-PE38	GPC3	PE38	586.0	100	Hep38B	
HN3-mPE24		PE24 with 5 point mutations to reduce B cell binding	592.0	99		(70)
HN3-T20		PE24 with 6 point mutations to reduce T cell binding	766.0	77		
HN3-T19		PE24 with 10 point mutations to reduce B and T cell binding	1082.0	54		

A similar approach was used to identify B cell epitopes in diphtheria toxin (71). Highly hydrophilic amino acids on the surface of the toxin were mutated, and the mutant constructs were injected into mice for screening. Constructs that did not activate the mouse immune system are speculated to be of low immunogenicity in humans as well (71). This approach, while simpler than the strategy used to generate LO10, suffers from the fact that mice and human have different self and non-self-selection, and immunogenic regions that activate a human immune system may not activate a mouse immune system.

### Deletion of Domain II of PE38

Protease evasion can reduce processing of the protein in the endosome and late endosome and therefore, reduce peptide presentation by MHC II molecules and T-cell activation. Weldon et al. found that domain II of PE38 was very sensitive to lysosomal protease digestion and furthermore that 102/113 amino acids in domain II can be removed without loss of activity as long as the furin cleavage site (in amino acids 274–284) remained (72) (Figure 1D). Deletion of the majority of domain II had the additional benefit of deletion of the immunogenic B and T cell epitopes in that domain. RITs with the resulting mutant toxin (designated LR for lysosome protease resistance) or PE24 (Table 2) were tested in three strains of mice and showed a greatly decreased antibody response (73).

### T Cell Epitopes

Elimination of B cell epitopes as described above should be effective in evading pre-existing antibodies. However, deletion of the immunodominant B cell epitopes cannot prevent B cells with low affinity B-cell-receptors from undergoing affinity maturation and class switching. These processes are supported by professional antigen presenting cells and helper T cells (58, 74). Unlike B cells, T-cell receptor specificity, does not change on antigen encounter. Once T-cell epitopes are eliminated, formation of new specificities is not expected (75). In a proof of concept study, the murine T cell epitopes in PE38 were mapped using a peptide library and IL2 ELISpot of immunized mice spleens. Alanine scanning of each amino acid within 15 mer epitopes revealed single point mutations that can prevent the T cell response. A new RIT was constructed with several point mutations in PE38 that were effective on preventing anti PE antibodies and Nabs (76). Additional studies in BALB/c mice reinforced the identification of a subdominant murine T cell epitope in domain III (77). This study also showed that a slightly modified version of the de-immunized PE (A505H) using a different mode of administration and adjuvant has a significantly lower immunogenicity compared to PE24.

The human T cell epitopes in PE38 mapped using PBMCs from 50 donors that share similar HLA to the typical patient population in the western world. The PBMC were expanded with PE38 to allow antigen processing and presentation and enrich the T cells that recognize PE38 epitopes (78). The enriched T cells were re-stimulated with over-lapping peptides that span the sequence of PE38. T cell activation was monitored d using IL-2 ELISpot (79). IL-2 supports T-cell activation, differentiation, and memory and is a less specialized cytokine than IL-4 or IFN-γ (80). Twenty-three peptides whose sequence overlap had positive responses and made up eight T cell epitopes (65). One of these epitopes, located in domain II, was present in 21/50 donors (79). The eight T cell epitopes identified in naïve donor PBMC were also identified using samples from 16 cancer patients previously treated with PE38 containing RITs and who had mounted an immune response to the protein. This supports the conclusion that PE38 has eight T cell epitopes and other regions of the protein are less immunogenic. Interestingly, similar assays using PBMCs from immunized HCL patients show several epitopes missing (65). Further work will address the absence of some epitopes in HCL patients and why cells recognizing these epitopes are absent in these patients.

**Figure 4:**
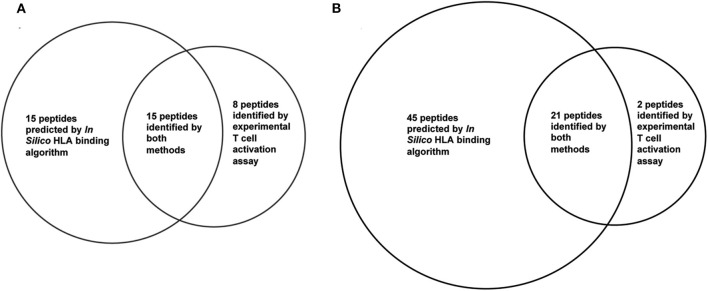
Overlap of experimental T cell epitopes and *in silico* HLA binding prediction. Twenty three T cell epitope were identified in PE38 by experimental T cell activation assays using 50 PBMC donors. *In silico* predicted binders in PE38 were predicted using the IEDB consensus HLA class II binding algorithm and 15 HLA-DR alleles. A peptide was considered a potential epitope using a threshold of **(A)** top 30 predicted binders and **(B)** top 56 predicted binders (50%). Peptides 8 and 9 were false negatively predicted using both thresholds.

HLA binding algorithms that predict the binding affinity of peptides to polymorphic HLA II molecules can be used to predict or narrow down peptides for potential T-cell epitopes. Overpredictions are expected for such algorithm predicted epitopes due to various factors involved in T-cell activation that cannot be predicted by the HLA binding, including antigen processing in the endosome, T-cell receptor binding, and T-cell activation. To compare the experimentally identified epitopes with *in silico* predicted epitopes, two primary HLA binding algorithms: Propred (81) and IEDB Consensus (82) were used to predict promiscuous binding to 15 common HLA DR alleles (83). Venn diagrams showing comparison of the predicted peptides using the *in-silico* analysis and the experimental approach is shown in Figure 4. The top 30 stringently predicted peptides had an overlap of 15 peptides with the 23 experimental peptides. This left 8 peptides (representing four epitopes) mis-identified by the analysis as negative. A less stringent threshold of 56 peptides (choosing 50% of the peptides as positive) had a much better precision and predicted 21 of the 23 peptides. However, the epitope in peptides 8 and 9 was overlooked by the algorithm. While overpredictions are expected, underpredictions are not, and they such reduce the effectiveness of these computational tools for prediction of T-cell epitopes. HLA binding inhibition assays revealed that the missed epitope in peptides 8 and 9 was solely presented by HLA DP presentation molecules and not DR (84). The algorithm could not have predicted binding to this peptide, because the query was limited to DR alleles. Interestingly, re-analysis of the HLA binding prediction by adding 8 DP alleles still failed to recognize this epitope as a strong binder (84). This indicates that HLA binding algorithms cannot accurately predict all T-cell epitopes and should always be validated with experimental work.

The epitopes in domain II of PE38 were eliminated by deletion of the whole domain, except for the 11 amino acid furin cleavage site, which does not contain an epitope. To modify the epitopes in domain III, alanine scanning was used to identify amino acids that impact the T-cell epitopes. To ensure that the point mutation did not introduce a new T cell epitope PBMCs were stimulated with the mutant RIT and re-stimulated with the mutant peptides. Two of the epitopes (epitopes 2 and 6) were difficult to solve by alanine scanning, because the mutations caused loss in activity. To aid with that, Rosetta computational protein design methods was combined with an HLA binding algorithm to identify mutations that disrupt the binding to HLA II molecule and as the same time still maintained cytotoxic activity (85). Epitope 2 (in domain III) was not resolved using a single point mutation and required a combination of two-point mutations to diminish the T cell responses significantly (R494A and R505A). However, the cytotoxic activity was reduced 2- to 3-fold by one of these mutations (Table 2).

The six point mutations designed to remove of suppress T cell epitopes were combined into new RITs. LMB-T18 targets CD22 (65), LMB-T20 (Figure 1G), targets mesothelin, HN3-T20 targets GP3 (70), and LMB-273 targets BCMA (69). Each protein contains the mutated toxin as shown in Table 2. Re-analysis of LMB-T20 for T cell activation showed that cryptic or new epitopes did not emerge as a result of altered antigen processing in LMB-T20 (86).

Interestingly, when the de-immunized toxin used to make a RIT that targets human CD25 to kill human T-cell malignancies, the deletion of domain II significantly impaired the cytotoxic activity (67, 68). The dependency on domain II for cytotoxic activity is receptor specific and probably attributable to a variable internalization pathway. To improve the cytotoxic activity of CD25-targeting immunotoxin, PE38 was de-immunized with three more mutations in domain II (Table 2).

### B and T Cell De-immunized Immunotoxin

Intriguingly, two of the mutations intended to eliminate T cell epitopes are the same mutations that diminished binding to B cell epitopes. Both (R505A and R427A) have very high accessible surface area (150 and 142 Å, respectively) indicating these arginines are located on the surface of the molecule. Since B cell epitopes are known to contain bulky hydrophilic amino acids like arginine (87–89), it is not surprising that mutations that diminish T cell epitopes also diminish B cell epitopes. Other reports have shown that important epitopes may be shared by B and T cells (90–92), and a functional link between B and T cells that recognize overlapping peptides has been suggested (93).

To reduce reactivity with both B and T-cells, the mutations that eliminated T- and B-cell epitopes were incorporated into a single RIT that targets mesothelin (66). The final RIT (LMB-T14) (Figure 1H) has good cytotoxic and anti-tumor activity vs. human cell lines, patient-derived cells, and mouse tumor models. LMB-14 has reduced binding to serum from patients who developed antibodies compared to its unmutated parental immunotoxin. Unexpectedly, remapping of T-cell epitopes of LMB-T14 revealed that two mutations, that were introduced to eliminate conformational B-cell epitope, created a new T-cell epitope. This demonstrates the challenging balance between cytotoxic activity, B-cell and T-cell reactivity during de-immunization (66).

### Translation of De-immunization Effort (Immunogenicity of LMB-100)

The effectivity of T cell de-immunization efforts has not yet been tested in clinical settings. However, LMB-100, a B cell de-immunized RIT, has been tested in a recent trial.

It is difficult to compare the immunogenicity rate in this study to previous ones due to significant variation in the immunogenicity monitoring assays. While the immunogenicity response against SS1P was mostly monitored using a functional Nab assay, immunogenicity response against LMB-100 was monitored using an ADA bridge ELISA. Furthermore, blood half time concentration cannot be compared due to differences in dose, size and structure that can impact half time regardless of immunogenicity. Lastly, the clinical design of the SS1P study excluded patients who had elevated pre-existing antibodies to SS1P, presumably due to prior exposure to *Pseudomonas aeruginosa*, while 9/20 patients in the LMB-100 trial had pre-existing ADA.

To try and compare SS1P and its de-immunized counterpart, Alewine et al. compared the number of patients with effective RIT C_max_ levels (>100 ng/ml). They noted that more than half of patients receiving a second cycle of LMB-100 had detectable plasma drug concentrations. These results compare favorably with SS1P, for which more than 90% of the patients had undetectable drug levels by the start of cycle 2, after excluding patients with high preexisting antibodies (94, 95). This clearly indicates that LMB-100 de-immunization decreased the impact of immunogenicity. However, this improvement was only enough to allow one additional dose on the second cycle for most patients. Only a single patient was ADA negative after the completion of the therapy. We conclude that humanization of the antibody and silencing of the B cell epitopes (and some of the T cell epitopes) is helpful, but not sufficient to completely prevent an immune response. Future work is required to evaluate if the complete T cell de-immunized molecules (LMB-T20 and LMB-T14) are more effective in diminishing the immune response.

## Summary and Conclusion

In this review, we described various methods to monitor the immune response against RITs and efforts made to minimize the immunogenicity response in patients by combination therapy or rational design. LMB-100 is the first humanized and de-immunized RIT that was rationally designed for reduced B cell epitopes and evaluated in patient. Although it showed lower rates of immunogenicity compared to its parental RIT (SSIP), formation of ADA and Nab was delayed but not eradicated. Future work will require evaluation of novel approaches like elimination of both the B and T cell epitopes or combination therapy of immune suppressive agents and the de-immunized RIT.

## Author Contributions

All authors listed have made a substantial, direct and intellectual contribution to the work, and approved it for publication.

## Conflict of Interest

Drs. IP and RM are inventors on patents describing how to make less immunogenic immunotoxins. These patents have all been assigned to NIH.
